# Normative data for the segmental acquisition of contact heat evoked potentials in cervical dermatomes

**DOI:** 10.1038/srep34660

**Published:** 2016-10-06

**Authors:** Catherine R. Jutzeler, Jan Rosner, Janosch Rinert, John L. K. Kramer, Armin Curt

**Affiliations:** 1 Spinal Cord Injury Center, University Hospital Balgrist, University of Zurich, Zurich, Switzerland; 2 ICORD, University of British Columbia, Vancouver, British Columbia, Canada; 3 School of Kinesiology, University of British Columbia, Vancouver, BC, Canada

## Abstract

Contact heat evoked potentials (CHEPs) represent a neurophysiological approach to assess conduction in the spinothalamic tract. The aim of this study was to establish normative values of CHEPs acquired from cervical dermatomes (C4, C6, C8) and examine the potential confounds of age, sex, and height. 101 (49 male) healthy subjects of three different age groups (18–40, 41–60, and 61–80 years) were recruited. Normal (NB, 35–52 °C) followed by increased (IB, 42–52 °C) baseline stimulation protocols were employed to record CHEPs. Multi-variate linear models were used to investigate the effect of age, sex, and height on the CHEPs parameters (i.e., N2 latency, N2P2 amplitude, rating of perceived intensity). Compared to NB, IB stimulation reduced latency jitter within subjects, yielding larger N2P2 amplitudes, and decreased inter-subject N2 latency variability. Age was associated with reduced N2P2 amplitude and prolonged N2 latency. After controlling for height, male subjects had significantly longer N2 latencies than females during IB stimulation. The study provides normative CHEPs data in a large cohort of healthy subjects from segmentally examined cervical dermatomes. Age and sex were identified as important factors contributing to N2 latency and N2P2 amplitude. The normative data will improve the diagnosis of spinal cord pathologies.

Sensory deficits are a hallmark of a variety of neurological conditions. As a complementary tool to conventional clinical testing, laser and contact heat evoked potentials (LEPs and CHEPs, respectively) have been adopted as objective measures of small diameter afferents^1^,^2^,^3^,^4^,^5^,^6^,^7^,^8^,^9^,^10^. These investigations have primarily focused on examining areas of the body commonly associated with sensory impairments, including the face (e.g., trigeminal neuralgia), trunk and back (e.g., post-herpetic neuralgia), and distal parts of the upper and lower extremities (e.g., diabetic neuropathy)^11^,^12^,^13^,^14^,^15^. Accordingly, normative values have been well established for these areas^3^,^11^,^16^,^17^.

The approach of examining a general cutaneous area is suboptimal for the assessment of damage associated with a discrete spinal cord level (e.g., spinal cord injuries). In this case, a more “segmental” approach, involving a level-by-level assessment is needed. Such a segmental approach crucially requires examining dermatomes above, at, and below the level of injury in well-defined regions of the body innervated by individual spinal segments.

The goal of the present study was to apply an advanced testing protocol for establishing normative values of CHEPs form cervical dermatomes. These values are imperative to accurately determine when a recording in a patient with suspected spinal cord disorder (traumatic and non-traumatic) can be deemed “pathological”, and to assess potential confounding variables, such as height, age, and psychological factors. To this end, we examined CHEPs following thermal stimulation of low and high intensity in a large cohort of healthy individuals using standard anatomical locations corresponding to cervical (C4, C6, and C8) spinal segments.

## Material and Methods

### Subjects

A total of 52 female and 53 male healthy control individuals were recruited. Inclusion criteria were (1) age between 18–80 years and (2) native language either English or German. We specifically recruited individuals of different age groups (group 1: 18–40 years, Group 2: 41–60years, Group 3: 61–80 years). Exclusion criteria comprised pregnancy, intake of any medication (except birth control), and any obvious neurological condition. All participants provided written informed consent and all procedures described below were in accordance with the Declaration of Helsinki and approved by the local ethics board ‘Kantonale Ethikkommission Zürich, KEK’ (ref. number: EK-04/2006, cinicaltrial.gov number: NCT02138344).

### Study protocol

Prior to the measurement of CHEPs, all individuals were interviewed to assess pain catastrophizing using the German or English version of the pain catastrophizing scale (PCS)^18^. The PCS assesses individuals catastrophic thinking related to painful experiences. The overall reliability and validity of the PCS was demonstrated in earlier studies^19^,^20^,^21^,^22^.

**Figure 1:**
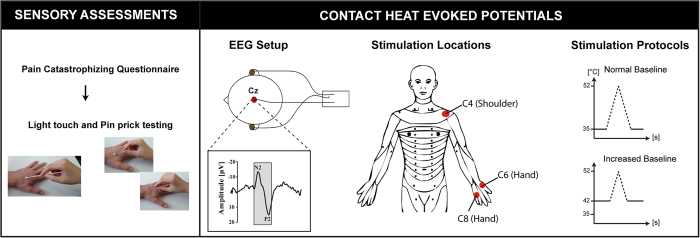
Study design. Prior to the recording of contact heat evoked potentials (CHEPs), all participants completed the pain catastrophizing questionnaire followed by pin prick and light touch testing. CHEPs were recorded from dermatomes C4, C6, and C8 (in a randomized order) employing normal followed by increased baseline stimulation.

As illustrated in Fig. 1, the study protocol consisted of unilateral assessments of pinprick and light touch followed by the recording of CHEPs. Three body parts in the distribution of the 4^th^ (C4, shoulder), 6^th^ (C6, dorsum of hand), and 8^th^ cervical segment (C8, dorsum of hand) were examined. The rationale for stimulating the dorsum of the hand was to stimulate the most distal part of the dermatome. Previous studies have shown that the separation of C6 and C8 dermatomes is best at the hand^23^. Furthermore, within the hairy skin of primates (i.e., dorsum) two distinct A-delta fibre receptor classes have been described and are almost equally distributed^24^. The type II AMH receptor mediates the first pain to heat stimuli, and is important for the fast transduction of contact heat stimuli. Evidence for the presence of type II-AMH in glabrous skin is sparse, and consequently it is difficult to elicit CHEPs stimulating these areas. Lastly, the contact surface of the chosen areas is sufficient allowing for a defined and evenly-distributed activation of a cutaneous area. The order of examination and body site tested was randomized for each subject^25^. Following light touch and pinprick testing, contact heat evoked potentials were recorded employing the conventional *normal baseline protocol* followed by the *increased baseline protocol.* The difference between the protocols was that the contact heat stimuli were delivered from either 35 °C (*normal baseline protocol*) or 42 °C (*increased baseline protocol*), respectively, to a peak temperature of 52 °C^5^,^26^. Contact heat evoked potentials (CHEPs) were recorded while subjects were lying in a supine position, with eyes open. In order to minimize ocular artefacts, subjects were asked to fix on a point on the ceiling, and to remain relaxed and quiet during testing. Prior to the testing, all individuals underwent a familiarization procedure that comprised of the presentation of two stimuli at the non-tested hand. A total of 15 contact heat stimuli per protocol (i.e., normal and increase baseline) were applied with an inter-stimulus time interval that randomly varied between 8 and 12 s. Traces contaminated with muscle or ocular artefacts were excluded in real-time and additional stimuli were applied in order to record 15 artefact-free traces. Cued by an auditory signal, individuals were asked to rate the perceived intensity of each stimulus from 0 (no pain) to 10 (most unbearable pain) two seconds after contact heat stimuli was delivered. The thermode was lightly repositioned subsequent to each stimulus within the dermatome tested to avoid peripheral sensitization/habituation (an area of approximately 4 × 4 cm).

### Contact Heat Evoked Potentials: Stimulating device and recording set up

To examine responses to noxious thermal stimulation, a contact heat stimulator was employed (Pathway, Medoc, RamatYishai, Israel). The thermode surface (diameter: 27 mm) consists of a heating thermo-foil covered with a layer of thermos-conductive plastic. The nominal heating rate of this device is 70 °C/s (thermo-foil), with a cooling rate of 40 °C/s (peltier element).

Cortical responses to the noxious heat stimuli were recorded with 9 mm Ag/AgCl surface disc electrodes filled with conductive adhesive gel. Scalp recording sites were prepared with Nuprep (D.O. Weaver & Co. Aurora, CO) and alcohol. Cortical recording electrodes were positioned in accordance with the International 10–20 system^27^. Both, N2 and P2, were acquired from an active vertex recording electrode (Cz) referenced to linked earlobes (A1-A2). The rationale for a reduced electrode set up arose from the fact that consistent negative and positive potentials, labelled N2 and P2, were reliably detected at Cz in previous studies^10^,^28^,^29^,^30^,^31^,^32^. All signals were sampled at 2000 Hz using a preamplifier (20000x, bandpass filter 1–300 Hz, ALEA Solutions, Zurich, Switzerland). Data were recorded with 100 ms pre-trigger and a one second post-trigger in a customized program based on LabView (V1.43 CHEP, ALEA Solutions, Zurich, Switzerland). The N2P2 waveform was visually detected based on the average of the 15-recorded trials. Waveforms had to be larger than 10 uV in order to be considered. The problem with waveforms below 10 uV is that the signal-to-noise ratio is too low to distinguish the signal from the noise.

### Analysis and statistics

Statistical analyses were performed using the computing environment R version 2.14.0 for Windows. All data were tested for normal distribution using the Kolmogorov–Smirnov test. Bonferroni correction was used to account for multiple comparisons. Statistical significance was set at α = 0.05.

The means (±standard deviation, SD) of the N2 and P2 latency (ms), the N2P2 amplitude (μV), as well as the pain rating were determined for each stimulation protocol. A linear mixed model was used to examine the main effect of the different stimulation protocols and stimulation sites (i.e., dermatome C4, C6, and C8) as well as the interaction effect between the stimulation protocol and site. Random subject effects were included in this model.

Exploratory analysis of bivariate relationships between clinical (i.e., age, sex, height, and PCS) and CHEPs parameters (i.e., N2 and P2 latencies, N2P2 amplitude) were examined using Pearson correlation analyses. In a subsequent analysis, the effects of age, sex, and height on the N2P2 amplitude and N2 latencies of both stimulation protocols (i.e., normal and increased baseline) were examined using a multi-variate general linear mixed models (3 dependent variables, 3 levels). N2P2 amplitude or N2 latency of each dermatome (i.e., C4, C6, and C8) were set as dependent variables, while sex was included as a fixed factor and age and height as covariates.

## Results

### Subjects

Out of 105 healthy subjects enrolled in the study, four had to be excluded due to: (1) technical problems during data acquisition (n = 3) and (2) intolerance of the contact heat stimuli applied (n = 1). The remaining 101 subjects comprised of 49 men and 52 women (mean age 46.2 ± 16.5 years) with an average height of 172.8 ± 8.9 cm. Men were significantly taller than women (F = 61.2, p < 0.001). The mean score of the PCS questionnaire was 10.5 ± 9.3. There was no difference between male and females in PCS score.

### Main effects of stimulation protocol

**Figure 2:**
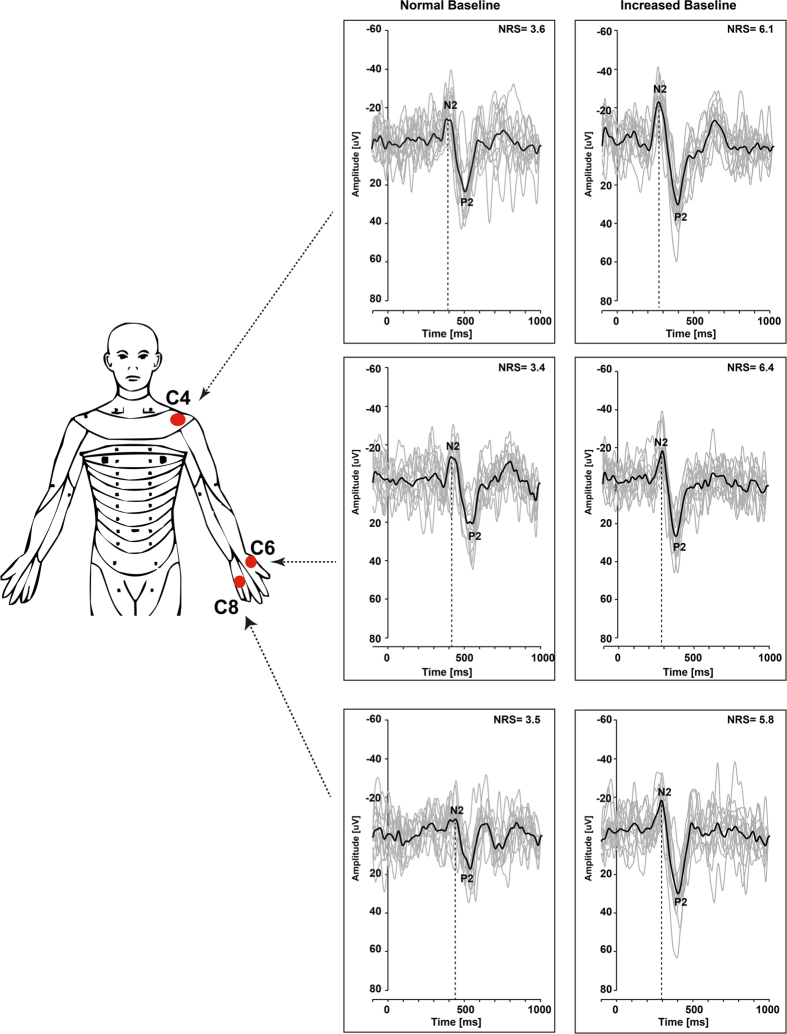
Representative example of CHEPs of each dermatome and both stimulation protocols.

A representative example of averaged CHEPs (N2/P2) of each dermatome (C4, C6, C8) and different stimulation protocols are illustrated in Fig. 2. Summary amplitudes, latencies for N2 and P2, and pain ratings both protocols, normal and increased baseline, are shown in Table 1. With the aim to record 15 artefact-free EEG traces, the total number of applied stimuli ranged between 15 and 22.

#### Effect of stimulation protocol, order, and site on CHEPs parameters

**Figure 3:**
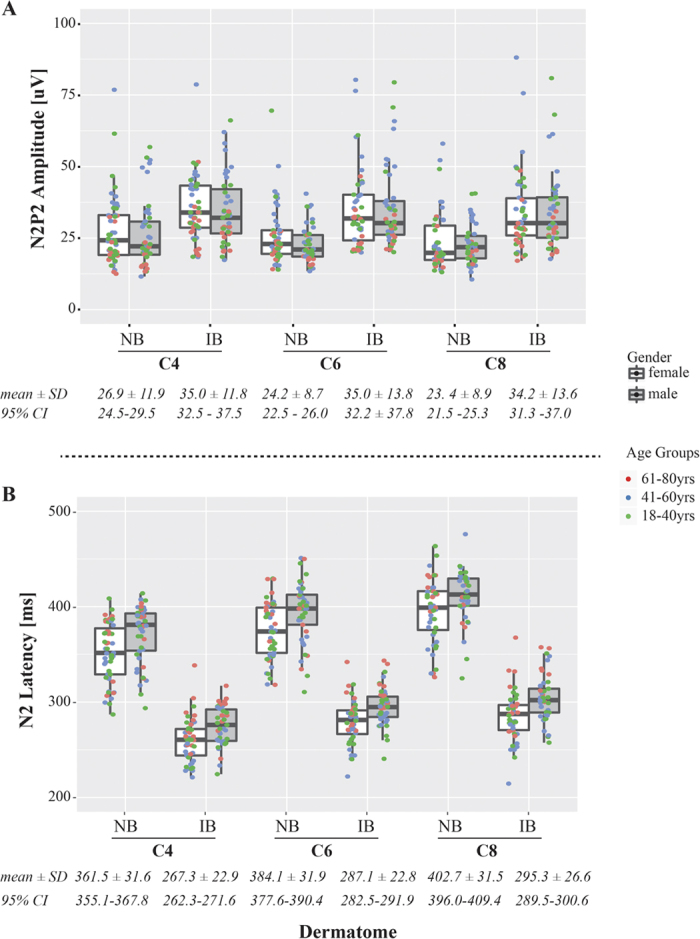
Summary of latencies and amplitudes for each dermatome and both stimulation modalities. Increased baseline resulted in (**A**) increased N2P2 amplitudes and (**B**) decreased N2 latencies across all dermatomes and age groups. The inter-subject variability of N2 can be minimized by employing increased baseline stimulation.

There was a significant main effect of stimulus protocol on N2P2 amplitude (F = 101.8, p < 0.001), N2 (F = 126.7, p < 0.001) and P2 latencies (F = 46.4, p < 0.001), as well as rating of perceived intensity (F = 81.7, p < 0.001). Notably, increasing the baseline temperature from 35 °C to 42 °C resulted in augmented N2P2 amplitudes, shortened N2 and P2 latencies, and elevated perceived intensity in all dermatomes. The main effect of stimulation site was significant (both stimulation protocols) for N2P2 amplitude (NB: F = 3.5, p = 0.44, IB: F = 0.127, p = 0.88), N2 (NB: F = 39.0, p < 0.001; IB: F = 33.5, p < 0.001) and P2 latencies (NB: F = 7.0, p = 0.001; IB: F = 8.5, p < 0.001) as well as rating of perceived intensity (NB: F = 7.9, p < 0.001: IB: F = 4.2, p = 0.16). Rating of perceived intensity (both stimulation protocols) and N2P2 amplitudes (normal baseline protocol) were significantly higher in dermatome C4 compared to dermatomes C6 and C8. For both stimulation protocols, N2 and P2 latencies gradually increased from dermatome C4 to C6 to C8 (Table 1 and Fig. 3). Group-wise comparisons revealed significant increased latencies for C6 and C8 when compared to C4 as well as an increased latency for C8 compared to C6.

### Effect of age, body height, and PCS score

**Figure 4:**
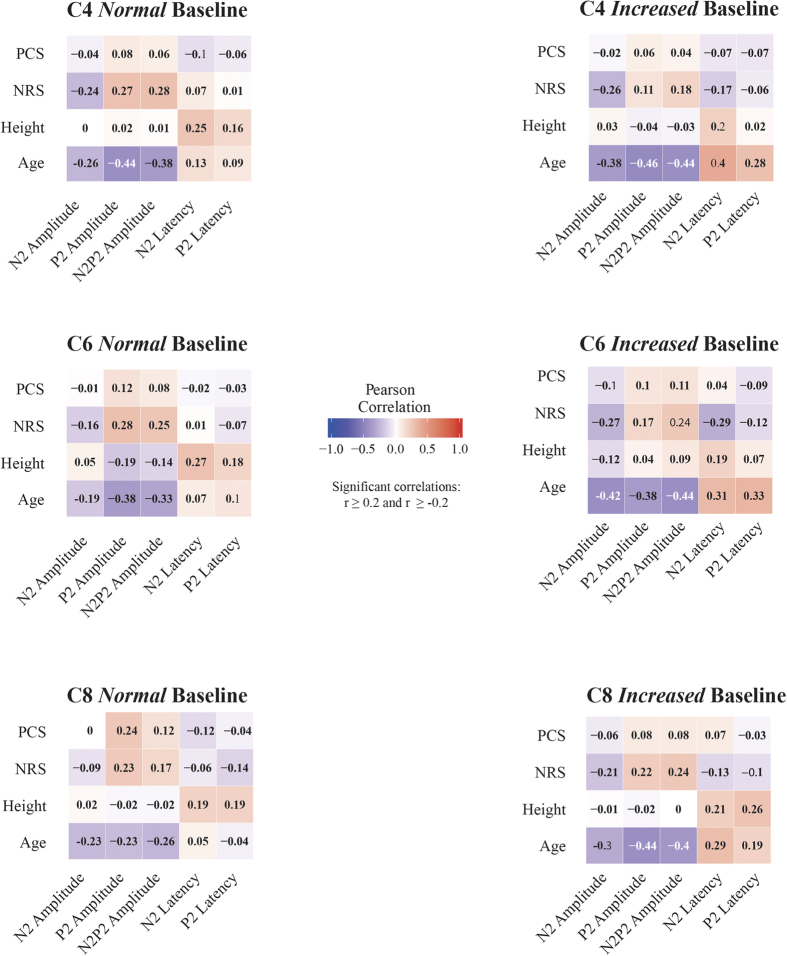
Pearson correlations between CHEPs parameter and demographics for each dermatome and stimulation protocol.

Pearson correlation analyses yielded significant negative correlations for age and N2P2 amplitude (C4NB: r = −0.376, p < 0.001; C4IB: r = −0.445, p < 0.001; C6NB: r = −0.331, p = 0.001; C6IB: r = −0.439, p < 0.001; C8NB: r = −0.264, p = 0.013; C8IB: r = −0.396, p < 0.001), N2 amplitude (C4NB: r = −0.256, p = 0.011; C4IB: r = −0.382, p < 0.001; C6IB: r = −0.415, p < 0.001; C8NB: r = −0.233, p = 0.030; C8IB: r = −0.296, p = 0.004), and P2 amplitude (C4NB: r = −0.435. p < 0.001; C4IB: r = −0.456. p < 0.001; C6NB: r = −0.382, p < 0.001; C6IB: r = −0.376, p < 0.001; C8NB: r = −0.234, p = 0.029; C8IB: r = −0.442, p < 0.001) for all dermatomes in both stimulation protocols. Age was further positively correlated with the N2 (C4IB: r = 0.399, p < 0.001; C6IB: r = 0.307, p = 0.003; C8IB: r = 0.294, p = 0.005) and P2 (C4IB: r = 0.284, p = 0.007; C6IB: r = 0.329, p = 0.001) latencies of the increased baseline protocol. Sex correlated with N2 latency (C4IB: r = 0.357, p = 0.001; C6IB: r = 0.363, p < 0.001; C8IB: r = 0.362, p < 0.001) of all dermatomes only when the increased baseline stimulation was employed. Lastly, height was correlated with N2 latency (C4NB: r = −0.246, p = 0.015; C4IB: r = 0.219, p = 0.038; C6NB: r = 0.269, p = 0.009; C6IB: r = 0.190, p = 0.066; C8NB: r = 0.195, p = 0.071; C8IB: r = 0.211, p = 0.045). All correlations between demographics and CHEPs parameters are summarized in Fig. 4.

The multivariate general linear models revealed a significant effect of age on N2P2 amplitude of all dermatomes in both stimulation protocols (C4NB: F = 10.2, p = 0.02; C4IB: F = 24.2, p < 0.001; C6NB: F = 10.5, p = 0.002; C6IB: F = 20.0, p < 0.001; C8NB: F = 6.7, p = 0.012; C8IB: F = 16.0, p < 0.001). Age (C4IB: F = 22.0, p < 0.001; C6IB: F = 12.6, p = 0.001; C8IB: F = 12.3, p = 0.001) and sex (C4IB: F = 3.3, p = 0.025; C6IB: F = 3.3, p = 0.024; C8IB: F = 3.3, p = 0.024) had a significant effect on the N2 latency when the increased baseline temperature protocol was employed.

## Discussion

The present study provides a protocol and normative data for the segmental assessment of cervical dermatomes with CHEPs. CHEP parameters were significantly associated with age and sex, but not psychological factors (i.e., PCS). Height, which was significantly associated with CHEPs according to a bivariate analysis, had no effect on CHEPs after accounting for sex and age. The use of an increased baseline stimulation protocol improved the acquisition of CHEPS by increasing N2P2 amplitudes and reducing N2 latency variability between subjects. The segmental approach described herein is well suited for the measurement of sensory deficits associated with a distinct level of damage in the cervical spinal cord.

### Correlations of age, sex, and height with CHEPs parameters

In the present study, age was negatively correlated with the N2P2 amplitude and positively associated with the N2 and P2 latencies. This confirms a number of previous LEP/CHEPs studies^11^,^17^,^33^,^34^. Changes of N2P2 amplitude as a function of age were evident in both stimulation protocols, normal and increased baseline. Demonstrating the sensitivity of our approach, the effect of age on N2 and P2 latencies was only evident using the increased baseline stimulation. Physiologically, age-related attenuation of amplitudes and prolongation of latencies have been attributed to a dysfunction of peripheral nociceptive mechanisms, alteration in CNS processing, or de-synchronization of the ascending nociceptive volleys^35^,^36^,^37^.

Also in agreement with previous studies, sex-related effects were observed for N2 and P2 latencies, and rating of perceived intensity^11^,^16^,^34^. Owing to longer afferent conduction distances, height is a plausible explanation for the sex-differences considering that male participants were significantly taller than females in our study. However, a multi-variable analysis indicates that sex, not height accounts for differences in CHEP latency. Previous findings regarding the effect of height on N2 latency are rather inconsistent, ranging from strong correlations to no effect^3^,^16^,^17^. Most studies, however, suggest that females report higher levels of perceived intensity to noxious heat stimulation than males^38^. Various biologic (e.g., skin thickness, brain structure), psychologic (e.g., anxiety and catastrophizing), and social factors (e.g., social expectations) have been proposed to account for sex related differences^39^,^40^,^41^,^42^,^43^. In the present cohort of healthy subjects, pain catastrophizing was comparable across age groups and sex. As such, the sex-related difference in ratings of perceived intensity is unlikely the result of greater catastrophizing in females.

No association between PCS and any of the CHEPs parameters (i.e., N2P2 amplitude, N2 and P2 latencies, and NRS) were identified. This contrasts with what has been reported for pain thresholds, which are significantly related to pain catastrophizing (i.e., higher PCS scores, lower pain thresholds)^44^,^45^,^46^,^47^. The lack of a relationship with PCS potentially indicates that CHEPs reflect underlying nociceptive processes that are independent of psychological factors.

### Mechanism of improved CHEP acquisition with increased baseline temperature

In line with our earlier investigations^5^,^48^, an increased baseline protocol (42 °C) notably improved the acquisition of CHEPs. Adopting a higher baseline temperature results in shorter stimulus duration and higher synchronization of peripheral small-diameter afferents^48^. The clear benefit of this protocol is larger amplitude N2P2 waveforms. Using a higher baseline temperature has previously revealed preserved sensory sparing in individuals with absent pinprick and/or no sensation to conventional contact heat stimulation^26^,^49^. Thus, in addition to defining a level of injury, this protocol is more sensitive to assess the severity of damage in cutaneous areas with reduced sensation.

In order to decrease latency jitter (i.e., increase synchronization), previous studies have applied contact heat close to the midline^11^,^50^,^51^. This approach, which decreases peripheral conduction distances, has two major limitations in terms of segmental assessment of patients with cervical spinal cord disorders. First, from an anatomical perspective, cutaneous areas corresponding to single spinal levels are less well defined at the midline^23^. Comparatively, dermatomal overlap is less pronounced distally^23^. Thus, testing cervical segments distally (i.e., C4 on the shoulder and C6/C8 on the hands) is more ideal for the segmental assessment of damage in the spinal cord. Second, stimulating along the midline, particularly along the back, may be difficult for some patients with limited mobility. For example, positioning an individual with a severe spinal cord injury on their stomach can be uncomfortable and require a considerable amount of assistance. Comparatively, testing the arm and hands allows a patient to be comfortably and safely positioned in a sitting or supine position. Taken together, incorporating the increase baseline stimulation in the segmental assessment provides an elegant and clinically feasible alternative to improve the acquisition of CHEPs without the need to test at the midline.

### Potential strategies to examine pathology

The goal of establishing normative CHEPs values in cervical dermatomes was primary but not limited for clinical applications in traumatic spinal cord injury and cervical spondylotic myelopathy. While traumatic spinal cord injury is a relatively rare event, diagnosing level of injury and monitoring changes near the level of lesion (i.e., syringomyelia) is of paramount importance^6^,^8^,^26^,^52^. Diagnosing the level of impairment and monitoring deteriorating neurological function is equally important in the case of cervical spondylotic myelopathy, for which surgical intervention may be indicated. Conventionally, sensory function following damage to the spinal cord is evaluated by testing light touch and pinprick sensation (i.e., International Standards for the Neurological Classification of SCI, ISNCSCI)^53^. However, the subjective nature (one has to rely on the patient’s appreciation) and classification with an ordinal scale limits their sensitivity to detect pathology. Sensory evoked potentials, such as CHEPs, constitute a complementary strategy to overcome these limitations based on objective and quantitative information (i.e., amplitudes and latencies). The overall reliability and validity of CHEPs was demonstrated previously^6^. Assessing multiple dermatomes along the spinal axis spanning the anatomical area of damage allows the precise determination of the level of lesion and potentially provides information about the injury severity. Moreover, clinical meaningful changes (recovery or aggravation of sensory function) occurring close to the injury level can be disclosed and rigorously tracked over time.

The question now turns to how CHEPs can be interpreted in a clinical setting. The difficulty with interpreting impairments based on amplitude is two-fold. First, there is a prominent effect of arousal and attention, well established in the literature^54^,^55^,^56^. Second, between-subject variability in amplitude is high, and this persists to for increased baseline temperature. For example, an objective cut-off to classify CHEPs as pathological would require, particularly in older male subjects, N2P2 amplitudes that are difficult to distinguish from background “noise”. Testing a contralateral side would overcome this limitation; however, spinal cord damage is often bilateral, so there may not always be a “normal” matching dermatome for comparison.

To overcome these problems, latency has been proposed as a more sensitive measure of pathology^17^. Compared to LEPs, however, the conventional acquisition of CHEPs remains disadvantaged by higher between-subject N2 latency variability^57^. Presumably, this is related to longer stimulus durations, which introduce heterogeneity in peripheral recruitment. Based on our observations, increasing the baseline temperature preceding contact heat stimulation has the significant potential to improve the clinical interpretation of evoked potentials. Indeed, adopting this protocol resulted in between-subject variance (Fig. 3) comparable to reported LEPs^17^. Therefore, this protocol not only improves the acquisition of CHEPs at the single subjects (i.e., increases amplitude and the likelihood to detect afferent sparing)^5^,^6^,^48^, but also interpretation at the group level.

### Limitations

A major limitation of our study is that normative values are limited to studies or clinical sites using the same CHEP acquisition equipment. Centers interested in adopting CHEPs will still need to develop their own normative data. Also, increased baseline stimulation may not be tolerated for some individuals. This was the case for two individuals in our study. However, we have previously demonstrated that increasing the baseline temperature and adjusting the peak stimulation temperature results in improved CHEP acquisition^48^. This approach could then be utilized in subjects that report high pain ratings to a fixed 52 °C stimulation.

## Conclusion

The current study provides normative CHEPs data in a large cohort of healthy subjects from segmentally examined cervical dermatomes (C4, C6, and C8). Age and sex were identified as important factors contributing to N2 latency and N2P2 amplitude variability. Employing an increased baseline stimulation protocol reduced latency jitter within subjects, yielding larger N2P2 amplitudes, and substantially decreased inter-subject N2 latency variability. The latter is important in terms of generalizing our findings to the interpretation of pathological CHEPs in patient populations. The normative data can be used to diagnose a variety of spinal cord pathologies, as well as track changes in sensory function emerging close to the level of lesion. The recovery of a patient but also the success of therapeutic intervention (e.g., surgery or drugs) can be monitored by the segmental assessment approach.

## Additional Information

**How to cite this article**: Jutzeler, C. R. *et al*. Normative data for the segmental acquisition of contact heat evoked potentials in cervical dermatomes. *Sci. Rep.* **6**, 34660; doi: 10.1038/srep34660 (2016).

## Figures and Tables

**Table 1:** Summary of thermal thresholds, CHEPs parameters (i.e., latencies, amplitudes), and pain rating of the study cohort.

Parameter	C4 Dermatome	C6 Dermatome	C8 Dermatome	
*Normal Baseline*	Tested dermatomes (N)*	100	99	98
	Light touch (Score 0–2)^¥^	2 ± 0 (2)	2 ± 0 (2)	2 ± 0 (2)
	Pinprick (Scores 0–2)	2 ± 0 (2)	2 ± 0 (2)	1.98 ± 0.14 (1–2)
	N2 Latency [ms]	361.5 ± 31.6 (287–414)	384.1 ± 31.9 (310.5–451)	402.7 ± 31.5 (325–476)
	P2 Latency [ms]	504.1 ± 45.0 (379.5–628)	526.5 ± 61.0 (396–672)	529.6 ± 45.9 (423–641.5)
	N2Amplitude [uV]	19.0 ± 6.4 (5.2–43.9)	11.5 ± 4.5 (4.5–33.0)	10.7 ± 5.4 (2.5–36.5)
	P2 Amplitude [uV]	14.0 ± 6.5 (5.1–32.9)	12.8 ± 5.2 (5.1–36.5)	12.7 ± 4.7 (5.5–30.0)
	N2P2 Amplitude [μV]	26.9 ± 11.9 (14.6–76.8)	24.2 ± 8.7 (13.5–69.5)	23. 4 ± 8.9 (10.5–58.0)
	Pain rating (NRS)	5.6 ± 1.9 (1.6–9.8)	4.9 ± 1.9 (0.5–9.5)	4.6 ± 2.0 (0.5–9.6)
	Tested dermatomes (N)^†^	99	100	100
*Increased Baseline*	N2 Latency [ms]	267.3 ± 22.9 (221–338.5)	287.1 ± 22.8 (222–343.5)	295.3 ± 26.6 (224.5–367.5)
	P2 Latency [ms]	267.2 ± 22.9 (221–338.5)	422.2 ± 44.8 (338–546.5)	428.2 ± 49.7 (304–598.5)
	N2Amplitude [uV]	18.4 ± 6.4 (8.5–44.2)	18.1 ± 7.6 (8.05–49.8)	17.6 ± 8.3 (6.69–49.9)
	P2 Amplitude [uV]	16.5 ± 6.5 (5.7–35.9)	16.9 ± 7.7 (6.09–42.9)	16.6 ± 6.7 (6.1–44.1)
	N2P2 Amplitude [μV]	35.0 ± 11.8 (19.3–78.7)	35.0 ± 13.8 (19.96–80.29)	34.2 ± 13.6 (17.1–88.1)
	Pain rating (NRS)	7.0 ± 1.7 (2.1–10)	6.3 ± 2.0 (2–9.9)	6.2 ± 2.1 (0.7–10)
